# CYP2S1 Knockout Promotes Intestinal Tumor Growth in *APC*^Min/+^ Mice and Its Clinical Significance

**DOI:** 10.7150/jca.111574

**Published:** 2025-07-10

**Authors:** Yaqing Du, Yunxia Kuang, Xiuqiong Meng, Bobing Zheng, Qinru Chen, Qian Yan, Jiangchao Li

**Affiliations:** 1 Laboratory of Oncology and Immunology, School of Basic Medical Sciences, Guangdong Pharmaceutical University, Guangzhou, 510006, China.; 2 Guangdong Institute of Gastroenterology, Guangdong Provincial Key Laboratory of Colorectal and Pelvic Floor Diseases, The Sixth Affiliated Hospital, Sun Yat-sen University, Room 703, Guangzhou, 510006, China.

**Keywords:** CYP2S1, Colorectal cancer, *APC* ^Min/+^, Beta-catenin, Transgenic mice.

## Abstract

Colorectal cancer (CRC) is the third most common cancer and the second leading cause of cancer-related death worldwide. Many studies have attempted to elucidate the role of cytochrome 450 (CYP450) polymorphisms in cancer susceptibility and tumor progression. However, the function of Cytochrome P450 Family 2 Subfamily S Member 1(CYP2S1), a member of the CYP450 family, in CRC remains unclear. Here, we constructed *APC*^Min/+^;CYP2S1^-/-^ mice. We found that CYP2S1 knockout in *APC*^Min/+^ mice led to an increased number of adenomas, accelerated tumor progression and enhanced proliferation and angiogenesis of adenomas. Consistently, *in vitro* experiments demonstrated that CYP2S1 silencing enhanced the proliferation, migration and invasion of CRC cells. Furthermore, CYP2S1 knockout promoted the nuclear translocation of β-catenin in intestinal epithelial cells of *APC^Min/+^* mice and in cancer cells, it also activated Wnt and modulated P53 signaling pathways, upregulating metastasis associated in colon cancer 1 (MACC1), which accelerated cancer cell proliferation and invasion. Database analysis revealed that CYP2S1 was upregulated in colorectal cancer and positively associated with better prognosis. In conclusion, these findings suggest that CYP2S1 represents a promising biomarker and therapeutic target for improving the prognosis and treatment of colorectal cancer.

## Introduction

Colorectal cancer is often diagnosed at intermediate to advanced stages. Among gastrointestinal cancers, colorectal cancer ranks highest in incidence, comprising about 10% of all cancer cases and exhibiting both high morbidity and mortality rates 1. In 2022, CRC was ranked third among worldwide diagnoses of malignancies, with an estimated 1.93 million newly diagnosed cases and 903,859 deaths reported annually 2. Researchers have adopted a combination of therapeutic approaches including chemotherapy, nutritional therapy, and immunotherapy to manage colorectal cancer. However, both the incidence and mortality rates of colorectal cancer continue to increase 3-5. Research has demonstrated that molecular profiling of colorectal cancer can enhance treatment strategies and extend survival by targeting the tumor's biological features in specific patient subgroups 6. Yet, the molecular mechanisms governing colorectal cancer development and metastatic progression remain incompletely understood. Therefore, there is a critical need for in-depth investigation of biomarkers associated with colorectal cancer to advance patient diagnosis and therapy.

*APC*^Min/+^ mice carry mutations in the *APC* gene, with 'Min' denoting 'multiple intestinal neoplasia' 7. This model recapitulates the development of intestinal tumors observed in familial adenomatous polyposis (FAP) patients, displaying multiple adenomas in the intestine. Consequently, it serves as a valuable tool for investigating FAP pathogenesis 8. Li et al. demonstrated that silencing the CYP2S1 gene activated the MAPK/ERK-AHR signaling axis and triggered the apoptotic program of cancer cells in the context of BRAF^V600E^ mutation, which significantly inhibited the proliferation, invasion, migration, and tumorigenicity of thyroid cancer cells 9. Knocking down CYP2S1 can elevate endogenous prostaglandin E2 (PGE2) levels, thereby enhancing cell proliferation by inhibiting β-catenin phosphorylation and activating β-catenin signaling 10. The cytochrome P450 (CYP) isozyme family modulates the activity of certain precancerous compounds and chemotherapeutic agents through catalyzing diverse reactions 11. CYP2S1 is typically found in epithelial-derived cancers and is primarily associated with lipid synthesis and metabolism 12, 13. Studies suggest that dioxins can trigger CYP2S1 expression via an AHR-dependent pathway. CYP2S1 may play a role in the metabolic activation of carcinogens 14. However, the specific function and mechanism of CYP2S1 in colorectal cancer remain elusive.

In this study, *APC*^Min/+^;CYP2S1^-/-^ mice were bred to investigate the number of intestinal tumors incidence and morphology in CYP2S1-deficient *APC*^Min/+^ mice. Our findings demonstrate that CYP2S1 knockout enhances tumor progression in *APC*^Min/+^ mice and elucidates its impact on colorectal cancer cell proliferation and migration. These results highlight CYP2S1 as a promising therapeutic target for enhancing the process of colorectal cancer, with the potential to enhance patient survival and quality of life.

## Materials and Methods

### Mouse model construction and genotype identification

*APC*^Min/+^ mice (Nanjing Jicui Yaokang Biotechnology Co., Ltd., China) and CYP2S1^-/-^ mice (purchased from Saiye Biotechnology Co., Ltd., China) were used in this study. *APC*^Min/+^;CYP2S1^-/-^ mice were obtained by crossing CYP2S1^-/-^ mice with* APC*^Min/+^ mice. All mice were raised in the SPF Experimental Animal Center of Guangdong Pharmaceutical University. All control group mice (wild-type , WT) were selected from among C57BL/6J male mice of the same age (purchased from Guangdong Medical Experimental Animal Center; license SYXK (Guangdong) 2022-0125). All animal experiments were approved by the Animal Ethics Committee of Guangdong Pharmaceutical University (Animal Ethics Approval No. Gdpulacspf (2021002-3)). Genomic DNA was extracted from mouse toe tissue with the alkaline lysis method and amplified by PCR, followed by agarose gel electrophoresis (Table S1).

### Hematoxylin-eosin staining and Immunohistochemistry

Tissue samples were fixed with 10% neutral formaldehyde, embedded in dehydrated paraffin, and sliced into 4 μm sections. The sections were dewaxed, hydrated, subjected to hematoxylin-eosin (H&E) staining, dehydrated with different gradients of ethanol solutions, made transparent, sealed with resin glue. After dewaxing and dehydration, the tissue sections were subjected to high-pressure antigen retrieval with 10 mmol/L sodium citrate buffer (pH 6.0). Next, the sections were incubated overnight at 4°C with primary antibodies, including anti-CYP2S1 (1:100, HUABIO, ER63173), anti-Ki67 (1:100, Abcam, ab16669, USA), anti-CD31 (1:100, Abcam, ab28364, USA). Next, sections were incubated with secondary antibodies for 1h at 37℃, and color was developed with DAB (8059S, CST, USA). The sections were counterstained with hematoxylin. Digital images were captured using a microscope (Olympus CX31, New York, USA).

### Immunofluorescence

The cells were seeded on slides, washed with PBS, fixed with paraformaldehyde, treated with 0.1% Triton X-100 in PBS, blocked with blocking solution, incubated with the corresponding primary antibodies (β-catenin, BOSTER, BM1766, 1:100) overnight at 4 °C, incubated with secondary antibodies in the dark for 1 h, restained with DAPI solution, and photographed under a Zeiss laser scanning confocal (LSM880). The images were processed using ZEN software and were analyzed for fluorescence intensity and counted with imageJ.

### Cell culture and transfection

The colon cancer cell lines NCM460, SW480, HCT116, HT29, SW620, DLD1 and RKO were obtained and maintained in our laboratory. Cells were routinely cultured in DMEM (Gibco) supplemented with 10% fetal bovine serum (Gibco) and 1% penicillin streptomycin (Sigma) and cultured in an incubator containing 5% CO_2_ at 37 °C. During the transfection of small interfering RNA (siRNA), the synthesized siRNA sequence of CYP2S1 (GenePharma Co., Ltd.) at a concentration of 100 nM and a control sequence were transfected into HT29 cells using liposome 2000 (Invitrogen, 2233883). After 48 h of transfection, the transfection efficiency of the siRNAs was measured by Western blot. The sequences of the siRNAs are listed in Table S2.

### Western blot

Cells or tissues were collected, and total protein was extracted with RIPA buffer. The protein sample was mixed with loading buffer and heated at 100 °C for 10 min. The protein sample was separated via 10% sodium dodecyl sulfate‒polyacrylamide gel electrophoresis (SDS-PAGE). The separated protein was transferred to a nitrocellulose membrane. Membranes were incubated overnight at 4 °C with primary antibodies, followed by incubation with HRP-conjugated secondary antibodies. The membranes were then incubated with secondary antibody at room temperature for 1 h and photographed after enhanced chemiluminescence (ECL) reagent development. The list of antibodies is shown in Table S3.

### Quantitative Real-Time PCR

Total mRNA was extracted using an RNA extraction kit and the RNA was subsequently reverse transcribed into cDNA by a reverse transcription kit following the manufacturers 'protocols. Finally, the mRNA expression of the target genes was detected by qPCR. The volume of the qPCR system was 20 μL, including 10 μL of 2× SYBR^TM^ Green qMix, 0.5 μL of forward primer (10 μM), 0.5 μL of reverse primer (10 μM), 1 μL of cNDA template, and 8 μL of nuclease-free ddH_2_O. The PCR procedure was as follows: predenaturation at 95 °C for 30 s, followed by 45 cycles of denaturation at 95 °C for 5 s, annealing at 60 °C for 30 s, and extension at 60 °C for 30 s. The primer sequences are listed in Table S1.

### Database analysis

TIMER2.0 (http://timer.comp-genomics.org) was used to obtain data on CYP2S1 expression in cancers. UALCAN (http://ualcan.path.uab.edu/index.html) was used to predict the gene expression distribution. Kaplan-Meier survival curves (https://kmplot.com) revealed that the expression of CYP2S1 was correlated with the prognosis of CRC patients. The Human Protein Atlas (https://www.proteinatlas.org) showed the expression levels of CYP2S1 in different human organs and cells. GEPIA2 (http://gepia2.cancer-pku.cn) was used to analyze the expression level of CYP2S1 in tumor and normal colorectal tissues based on data from The Cancer Genome Atlas (TCGA).

### Methylene blue staining

The fixed intestinal tract was immersed in a methylene blue dye solution for 10-30 s. The samples were then differentiated with 70% ethanol until the intestinal tumors were clearly distinguishable from the surrounding normal tissue based on color contrast.

### CCK-8 cell proliferation assay

Cells were seeded into 96-well plates at a density of 2×10^3^ cells in 100 μL of medium. At the indicated time points, 10 μL of CCK-8 solution was added to each well, mixed gently, and incubated for 1-2 hours at 37 °C. The absorbance was then measured at 450 nm using a microplate reader. Cell proliferation was assessed by comparing the absorbance values among the experimental groups.

### Wound healing assay

When the cell density reached approximately 90%, a linear wound was created using a sterile 200 μL pipette tip, The samples were gently washed with sterile PBS and the medium was added to continue culture. Images of the wound area were captured at 0, 12, and 24 hours, and the scratch width of each well was measured using ImageJ software to assess cell migration.

### Colony formation assay

Well-grown cells were seeded into a 6-well plate at a density of 2×10^3^ cells per well of in 2 mL of complete medium. After mixing, the cells were continuously cultured for one week until obvious cell colony formation was observed. The cells were then washed twice with PBS, fixed with 4% paraformaldehyde for 10 minutes, and stained with crystal violet., Excess dye was removed by rinsing with water. After drying, using microscope to observation and photographing to record the number of colonies in the 6-well plate.

### Cell migration and invasion assays

Digested CRC cells were resuspended in serum-free medium. And the cell density was adjusted to 2.5×10^5^ cells/mL. Then, 200 μL of cell suspension was added to the upper chamber (with or without Matrigel), and 600 μL of complete medium was added to the lower chamber. After 24 h, the chamber was removed, and the cells were fixed with 4% paraformaldehyde for 10 min. The cells were dyed with 0.1% crystal violet for 20 min. Images were captured under a microscope, and the number of stained cells was quantified.

### RNA-seq sequencing

HT29 cells in the si-CYP2S1 group and si-NC group were collected and verified. Small RNA libraries were constructed and sequenced on the Illumina sequencing platform by Gene Denovo Biotechnology Co., Ltd. (Guangzhou, China).

### Statistical analysis

H&E, IHC and WB data were analyzed with ImageJ software. All experiments were repeated at least three times. Statistical analysis was performed using Prism 9 software. The data was expressed as the means ± standard deviation unless otherwise indicated. Student's t test was used for comparisons between two groups (two-tails distribution). A *P* value of less than 0.05 was considered statistically significant.

## Results

### CYP2S1 knockout promotes intestinal tumor growth in *APC*^Min/+^ mice

CYP2S1^+/+^ (wild-type) and CYP2S1^-/-^ (mutant) mice were identified with PCR (Figure S1A). Body weights of the male and female mice (n = 5 per group) were recorded weekly from the fourth week after birth. No significant difference in body weight was observed between CYP2S1-knockout and wild-type mice, indicating that CYP2S1 deletion did not affect growth (Figure S1B and S1C) We subsequently mated *APC*^Min/+^ mice with CYP2S1^-/-^ mice to construct an *APC*^Min/+^;CYP2S1^-/-^ hybrid mouse model. Genotypes were confirmed by PCR (Figure S1D and E).

**Figure 1:**
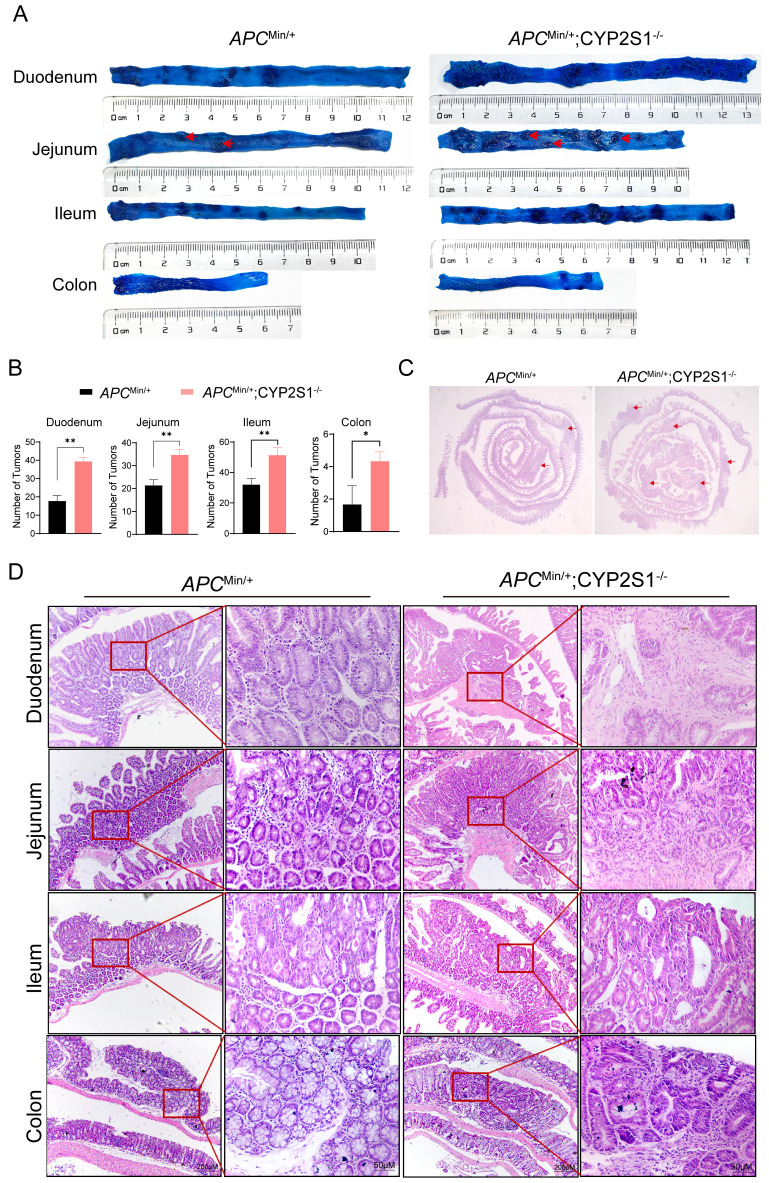
CYP2S1 knockout promotes intestinal tumorigenesis in *APC*^Min/+^ mice. (A and B) The number of adenomas (red arrows) in the duodenum, jejunum, ileum and colon of *APC*^Min/+^ mice and *APC*^Min/+^;CYP2S1-/- mice was analyzed by methylene blue staining (n = 3, *P<0.05, **P<0.01). (C) Changes (red arrows) in the morphology of the whole intestine of *APC*^Min/+^ mice and *APC*^Min/+^;CYP2S1-/- mice, as determined by H&E staining. (D) Morphological changes in various regions of the intestine in *APC*^Min/+^ mice and *APC*^Min/+^; CYP2S1-/- mice were observed by H&E staining (scale bar = 200μM; scale bar = 50μM).

The duodenum, jejunum, colon and ileum of *APC*^Min/+^ mice and *APC*^Min/+^;CYP2S1^-/-^ mice were stained with methylene blue to visualize intestinal adenomas. The results revealed that the number of adenomas in the *APC*^Min/+^;CYP2S1^-/-^ mice increased significantly compared to *APC*^Min/+^ mice(Figure 1A and B). Moreover, H&E staining was performed to observe histomorphological changes in the intestinal tissues. Compared with those of *APC*^Min/+^mice, the intestinal tracts of *APC*^Min/+^;CYP2S1^-/-^ mice presented obvious hyperplasia and more adenomas (Figure 1C). Histopathological analysis showed that the intestinal tissues of *APC*^Min/+^;CYP2S1^-/-^ mice exhibited typical pathological phenotypes such as fusion of the intestinal wall to form a co-mural structure, a significant increase in pathological nuclear pleomorphism of epithelial cells, and hyperplasia of the intestinal epithelial layer (Figure 1D). In summary, compared with those in *APC*^Min/+^mice, the number of intestinal adenomas in *APC*^Min/+^;CYP2S1^-/-^mice was more significantly increased, the intestinal structure was disordered, and hyperplasia occurred.

### CYP2S1 knockout induced nuclear accumulation of β-catenin in neoplastic cells

**Figure 2:**
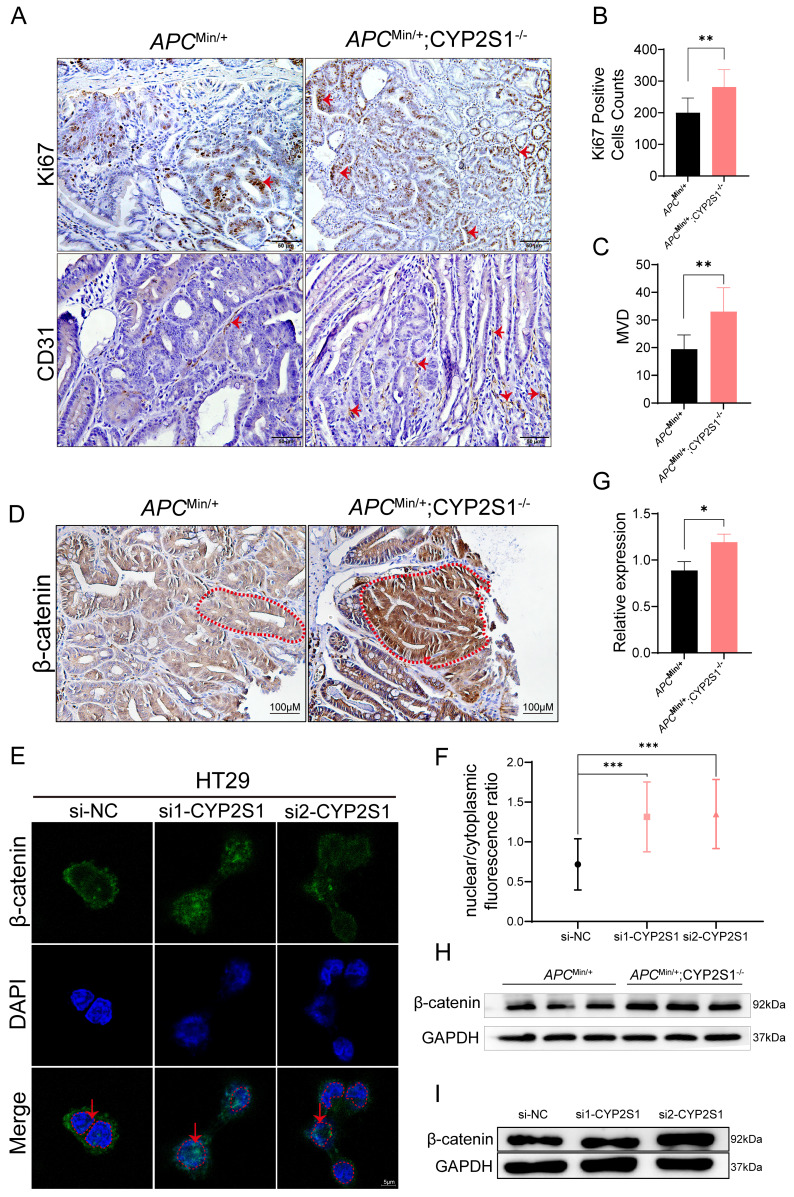
Increased accumulation of β-catenin in the nucleus after CYP2S1 silencing. (A)Ki67 and CD31 positive cells (red arrows) in the intestines of *APC*^Min/+^ mice and *APC*^Min/+^;CYP2S1-/- mice were detected by IHC (scale bar = 50μM). (B) The number of Ki67-positive cells was compared (n = 3, **P<0.01). (C) Comparison of the microvessel density between *APC*^Min/+^;CYP2S1-/- mice and *APC*^Min/+^ mice (n = 3, **P<0.01). (D) IHC detection of β-catenin expression in the intestines of *APC*^Min/+^ mice and *APC*^Min/+^;CYP2S1-/- mice. Red dashed lines indicat highlight the boundaries of intestinal glands (scale bar = 100μM). (E and F) Immunofluorescence staining revealed increased nuclear accumulation of β-catenin following CYP2S1 silencing. Red arrows indicate nuclear localization; red dashed circles outline nuclei (scale bar = 5μM, n = 12,***P<0.001). (G-H) Western blot analysis of β-catenin protein levels in the intestines of *APC*^Min/+^ mice and *APC*^Min/+^;CYP2S1-/- mice (n = 3, *P<0.01). (I) Western blotting was used to detect the protein expression level of β-catenin following CYP2S1 silencing in HT29 cells.

The above results revealed that both the volume and number of tumors were significantly increased and that the pathological features of the tumors further deteriorated. IHC staining further revealed that Ki67, a marker of cell proliferation, and CD31 (PECAM-1), a marker of angiogenesis, expression was significantly higher in *APC*^Min/+^;CYP2S1^-/-^ mice than in *APC*^Min/+^ mice (Figure 2A-C).

In *APC*^Min/+^ mice, *APC* mutation leads to the accumulation of β-catenin in the nucleus, thereby activating tumor-related transcription factors. Crossing CYP2S1 KO mice with *APC*^Min/+^ mice further led to increased nuclear β-catenin accumulation and significantly elevated intestinal β-catenin expression in *APC*^Min/+^;CYP2S1^-/-^ mice compared to *APC*^Min/+^ mice (Figure 2D). Furthermore, CYP2S1 silencing in HT29 cells led to increased nuclear translocation of β-catenin (Figure 2E and F) and the protein level was increased (Figure 2I). The protein expression level of β-catenin in the intestines of *APC*^Min/+^;CYP2S1^-/-^ mice increased compared to *APC*^Min/+^ mice (Figure 2G and H). These results indicated that CYP2S1 knockout in *APC*^Min/+^ mice enhanced intestinal adenoma development by promoting tumor cell proliferation, increasing angiogenesis, and facilitating nuclear translocation of β-catenin in colorectal cancer cells.

### Silencing CYP2S1 promotes colorectal cancer cell proliferation and invasion

**Figure 3:**
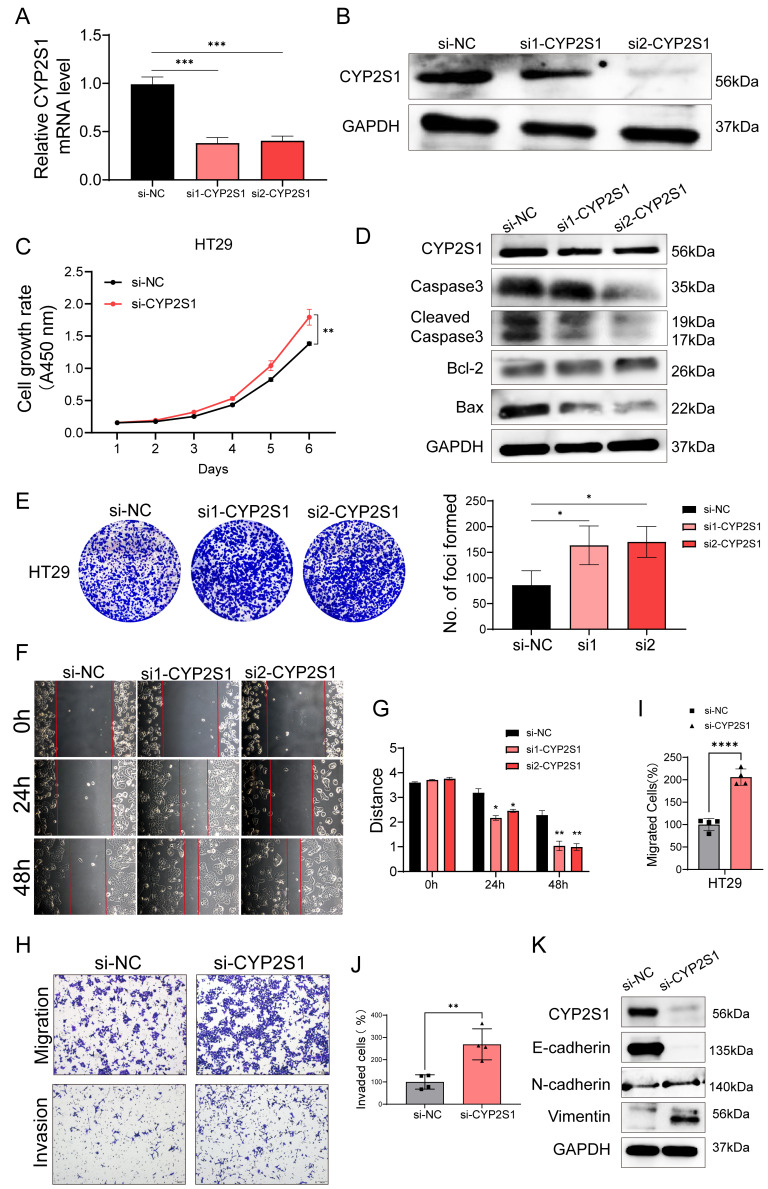
Silencing of CYP2S1 promotes growth, migration and invasion of colorectal cancer cells. (A)Validation of CYP2S1 mRNA silencing efficiency (***P < 0.001). (B) Western blotting was performed to verify the silencing efficiency at the protein level. (C) CCK-8 assay comparing the proliferation curves of HT29 cells with or without CYP2S1 silencing. (**P < 0.01). (D) Western blot analysis of Bax, Cleaved Caspase-3, Caspase-3 and Bcl-2 protein expression levels. (E) Colony formation assay revealed that silencing CYP2S1 increased the number of formed colonies by HT29 cells (*P < 0.05). (F and G) Wound healing assays demonstrated that the CYP2S1-silenced HT29 cell lines presented enhanced migration ability (*P < 0.05, **P < 0.01). (H-J) CYP2S1 silencing enhances cell migration and invasion (**P < 0.01, ****P < 0.0001). (K) Western blotting was performed to assess the expression levels of EMT-related proteins.

We selected highly expressed colorectal cancer HT29 cells for CYP2S1 silencing (Figure S2A-C). The mRNA and protein expression levels of CYP2S1 were lower in both the si1-CYP2S1 group and si2-CYP2S1 groups compared to si-NC group (Figure 3A and B). Both CCK8 and colony formation assays demonstrated that the si-CYP2S1 group exhibited enhanced proliferation compared to the si-NC group (Figure 3C and E). After silencing CYP2S1, the expression levels of pro-apoptotic proteins Bax, cleaved caspase-3, and caspase-3 were decreased, while the anti-apoptotic protein Bcl-2 was upregulated. (Figure 3D). These results suggested that silencing CYP2S1 promoted colorectal cancer cell proliferation by inhibiting apoptosis.

In addition, the wound healing distance in the si1-CYP2S1 and si2-CYP2S1 group was significantly greater than that in the si-NC group after 24 and 48 hours (Figure 3F and G). Transwell migration and invasion experiments further indicated that si-CYP2S1 group exhibited markedly increased cell migration and invasion compared to the si-NC group (Figure 3H-J). Compared with that in the si-NC group, the expression of E-cadherin in the si-CYP2S1 group was downregulated, while the expression of N-cadherin and vimentin was upregulated (Figure 3K) indicating enhanced epithelial-mesenchymal transition (EMT).

In summary, these *in vitro* results showed that CYP2S1 silencing promoted the proliferation, migration and invasion of colorectal cancer cells, consistent with above mouse experiments.

### CYP2S1 knockout upregulated MACC1 and modulated the P53 signaling pathway

**Figure 4:**
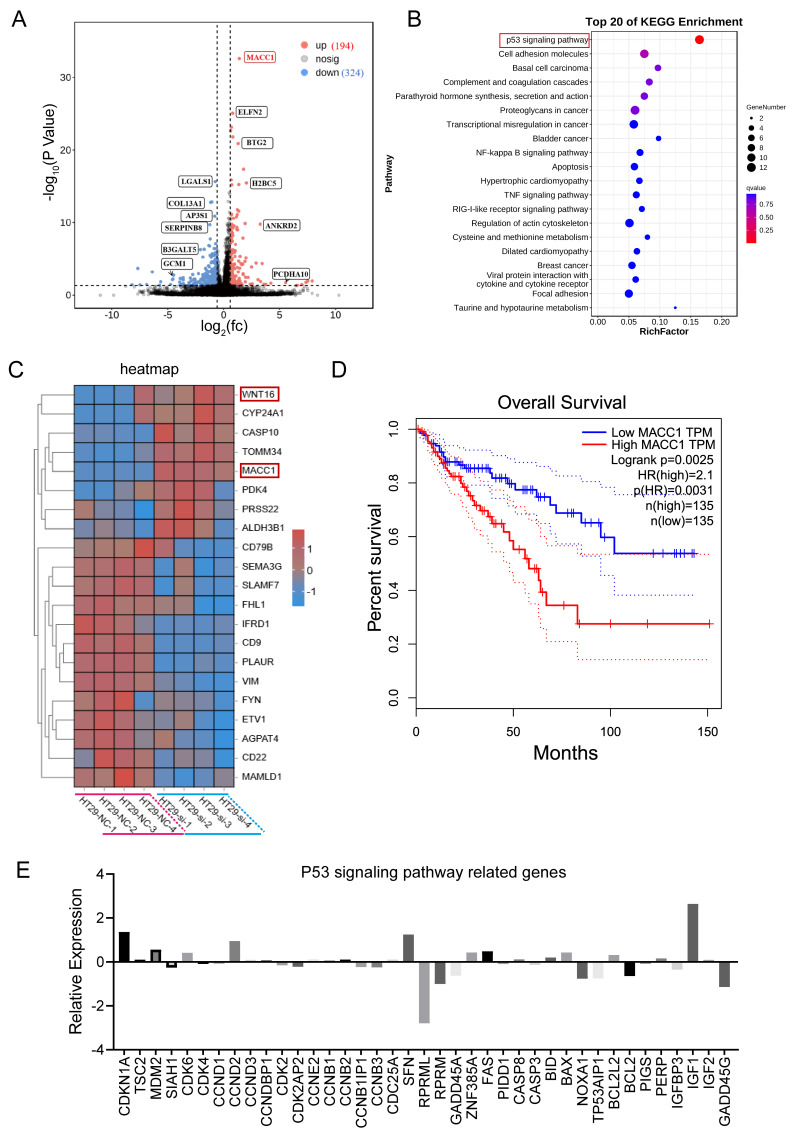
CYP2S1 knockout upregulates MACC1 and modulates the P53 signaling pathway. (A)Volcano plot of differentially expressed genes. (B) KEGG signaling pathway enrichment analysis. (C) Heatmap of differentially expressed genes between the CYP2S1-knockout and control groups. (D) Association between MACC1 expression and patient survival. (E) Relative expression levels of genes associated with the p53 signaling pathway after CYP2S1 silencing.

*In vivo*, CYP2S1 silencing enhanced the growth of intestinal adenomas and expedited their pathological progression. *In vitro*, silencing CYP2S1 increased the proliferation and migration of the colon cancer cell line HT29. To explore the molecular mechanism by which CYP2S1 knockout promotes tumor growth, RNA sequencing was performed. KEGG enrichment analysis revealed that many genes were enriched in the p53 signaling pathway. Data analysis revealed that a total of 518 genes exhibited significant differential expression. MACC1, ELFN2, BTG2, H2BC5, Wnt16, CYP24A1, CASP10, TOMM34 and other genes were significantly upregulated (Figure 4A-C). Among these gene changes in the RNA sequencing data, the correlation between CYP2S1 and β-catenin was analyzed. According to TIMER2.0 database, CTNNB1 (beta-catenin) was associated with BTG2 and MACC1 (Figure S3A and B). High expression levels of BTG2 and ELFN2 were associated with poor prognosis in CRC patients (Figure S3C and D). There was a linear relationship between CYP2S1 and ELFN2, WNT16 and LGALS1 (Figure S4A-C). Similarly, CTNNB1 expression showed positive correlations with ELFN2, WNT16, and LGALS1, although these associations were not statistically significant (Figure S4D-F).

**Figure 5:**
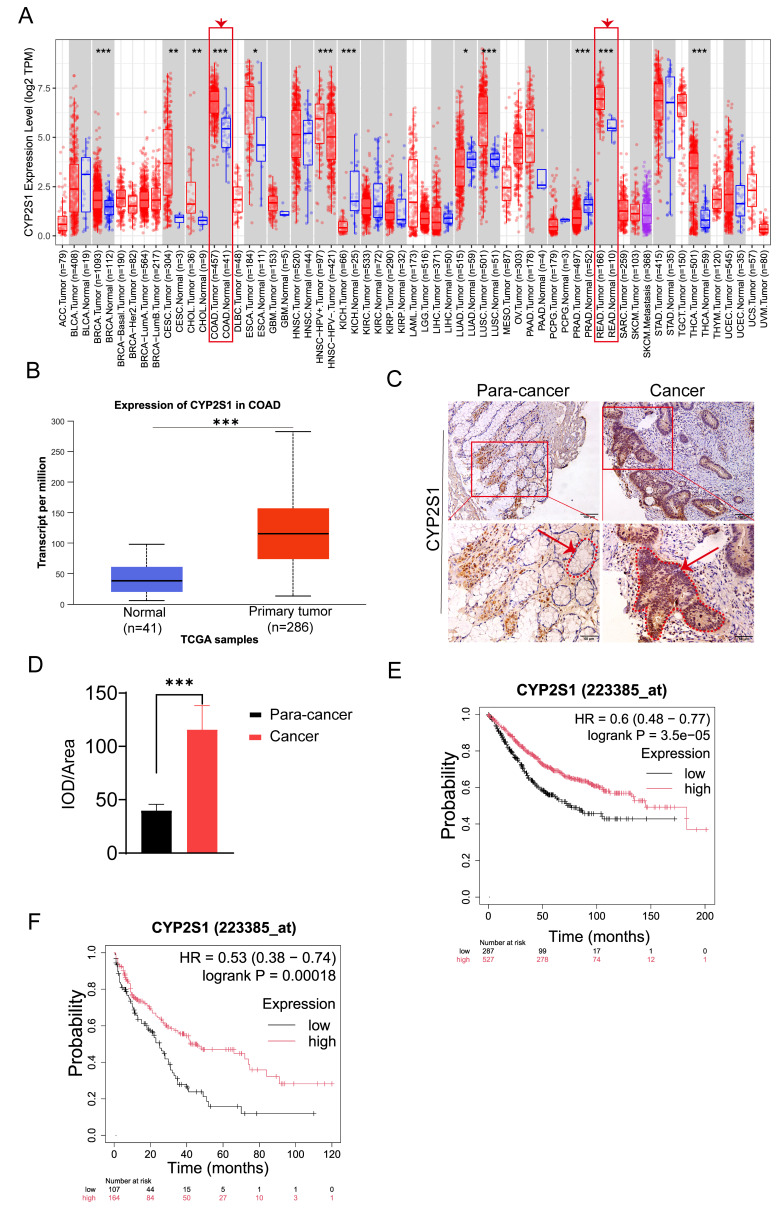
CYP2S1 is highly expressed in colorectal cancer, and its high expression is associated with better prognosis. (A and B) Analysis from both the TIMER 2.0 and UALCAN databases consistently demonstrates that CYP2S1 expression is significantly higher in colorectal cancer tissues compared to normal tissues (***P < 0.001). (C and D) Comparison of CYP2S1-positive cells in nontumor tissues and CRC tissues. Red arrows indicate the positions of intestinal glands, while red dashed lines delineate their boundaries (scale bar = 100μM; scale bar = 50μM,***P < 0.001). (E and F) Kaplan‒Meier survival analysis revealed that high expression of CYP2S1 was positively correlated with better prognosis.

KEGG enrichment analysis revealed that CYP2S1 mainly regulates the p53 signaling pathway, cell adhesion molecules, among others (Figure 4B). Survival analysis further demonstrated that high expression of MACC1 was associated with decreased overall survival in CRC patients (Figure 4D). In the P53 pathway several genes exhibited significantly expression differences between the CYP2S1-knockout group and the control group. Genes involved in cell cycle arrest and apoptosis were either upregulated or downregulated following CYP2S1 knockout. Notably, GADD45, which is involved in DNA repair and damage protection, was downregulated, while MDM2 expression was upregulated, and other p53-associated negative feedback regulators were downregulated (Figure 4E). These results indicate that CYP2S1 knockout accelerates tumor progression in CRC by disrupting P53 signaling and enhancing β-catenin activity. The bioinformatics data also revealed that the mutation in *APC*^Min/+^ accounted for 20.4% of CRC patients with CRC (Figure S5A) and that TP53 mutation accounted for 44.4% (Figure 5B). The two signaling pathways are affected at the same time, which aggravates the development and progression of CRC. Our experimental results are consistent with the clinical data.

### CYP2S1 expression was significantly correlated with the overall survival rate in cancer patients

The above data showed that CYP2S1 knockout promoted intestinal tumors growth both *in vitro* and *in vivo*. To clarify the clinical significance of CYP2S1, we evaluated its association with prognosis in cancer patients. Using the TIMER2.0 database, we found that CYP2S1 was significantly upregulated in colorectal cancer tissues compared to adjacent normal tissues. (Figure 5A). The finding was further confirmed by analysis using the UALCAN database (Figure 5B). IHC staining of paraffin sections of clinical tissue samples revealed that CYP2S1 expression was significantly higher in CRC tissues than in adjacent normal tissues (Figure 5C), with a statistically significant difference (Figure 5D, ***P < 0.001). These findings confirmed that CYP2S1 was highly expressed in CRC. Furthermore, Kaplan-Meier survival analysis demonstrated that high CYP2S1 expression was positively associated with better prognosis in patients with colorectal cancer (Figure 5E and F).

In addition, CYP2S1 expression was analyzed using the Human Protein Atlas database, which showed predominant expression in digestive tract tissues and cells (Figure S6A and B). IHC staining of tissues from 8-week-old C57BL/6J mice, including heart, liver, kidney, stomach, and colorectal samples, revealed CYP2S1 expression in gastrointestinal, lung, heart, liver, and kidney tissues, but not in immune organs such as the spleen, thymus, and lymph nodes. High expression was detected in gastric, digestive tract, intestinal and lung tissues (Figure S6C and D). Thess results were consistent with the database analysis.

## Discussion

Colorectal cancer is caused by a series of somatic genome changes that affect key driver genes 15.* APC*^Min/+^ mice are a common mouse model for the study of intestinal tumors 16. In this study, we constructed *APC*^Min/+^;CYP2S1^-/-^ hybrid mice to study colorectal cancer. Our results showed that CYP2S1 knockout promoted the occurrence and development of intestinal adenomas in *APC*^Min/+^ mice. A significant increase in the number of adenomas and an increase in cell proliferation and angiogenesis suggested that CYP2S1 knockout promoted the development of intestinal adenomas in mice.

The CYP450 superfamily is a group of more than 50 kinds of heme thioesterases that play important roles in maintaining physiological homeostasis 17. CYP2S1 belongs to the CYP450 family of monooxygenases 18. The cytochrome P450 monooxygenase pathway may play a key role in mediating the opposing effects of ω-6 PUFAs and ω-3 PUFAs on colorectal cancer 19. Metabolites of the ω-3 series of CYP450, especially DHA-derived EDP, can effectively inhibit angiogenesis, tumor growth and metastasis 20, 21.

Uncontrolled proliferation and abnormal differentiation of tumor cells, and their ability to invade and metastasize are the most basic biological characteristics of malignant tumors 22. Tumor growth and progression involve the destruction, invasion and metastasis of normal tissue structures 23. *In vitro*, we found that CYP2S1 knockout could increase proliferation and migration in CYP2S1-silenced HT29 cells, indicating that CYP2S1 deletion promotes the occurrence and development of colorectal cancer.

Some studies have found that abnormal changes in CYP expression are associated with poor prognosis in multiple cancers, including colon cancer 24, 25. Therefore, it is preliminarily determined that CYP acts as a cancer-promoting component in colon cancer. However, our *ex vivo* and *in vivo* experiments confirmed that CYP2S1 silencing promotes colorectal cancer, suggesting that CYP2S1 may be a cancer suppressor gene. So different CYP450 families need to be verified by more experiments.

In this study, RNA sequencing was used to analyze the gene changes. The specific expression of Wnt16 was significantly increased, and KEGG enrichment analysis revealed that it was mainly enriched in the P53 signaling pathway. Numerous scientific studies have confirmed that excessive activation of Wnt signaling is the leading cause of cancer in most human malignancies, including CRC 26, 27. The Wnt signal transduction pathway (Wnt pathway for short) mainly includes mainly the canonical Wnt/β-catenin signaling pathway and the noncanonical Wnt signaling pathways (the Wnt/planar cell polarity pathway and the Wnt/Ca^2+^ pathway) 28. The Wnt/β-catenin signaling pathway is generally activated by the binding of extracellular Wnt ligands to membrane receptors by autocrine or paracrine methods. The activated Wnt/β-catenin signaling pathway can induce changes in the stability of β-catenin and then transfer it to the nucleus, thereby promoting the expression of genes related to cell proliferation, survival, differentiation and migration 29. The stability and nuclear translocation of Wnt-mediated β-catenin is the key mechanism that promotes tumor cell proliferation and tumorigenesis 30. Wnt16b secretion promotes epithelial-to-mesenchymal transition (EMT) and reduces intercellular adhesion to increase tumor invasiveness 31. Studies have shown that metformin attenuates stemness and EMT in CRC cells by inhibiting the Wnt3a/β-catenin pathway 32. All the above studies support our data.

Meanwhile, we found that CYP2S1 silencing affected the expression levels of EMT-related protein. The p53 gene is located on the short arm of chromosome 17 and encodes a protein that regulates the cell cycle, DNA repair, senescence and apoptosis 33. P53 plays a central role in mediating cell responses, so its expression is strictly regulated 34. Loss of the p53-mediated apoptosis pathway is an important determinant of the progression from adenoma to malignant tumors. The main reason is that loss of p53 function enhances high cell proliferation activity and an uncontrolled cell cycle, leading to a key step in the development of colorectal cancer 35. Previous studies have shown that Wnt16b secretion usually regulates the tumor suppressor p53 in response to DNA damage and is critical for the beginning of aging 36. Our sequencing data revealed that KEGG pathways were enriched mainly in the p53 signaling pathway after CYP2S1 deletion. We speculate that the loss of CYP2S1 causes the inactivation of transcription genes in the p53 signaling pathway, resulting in an uncontrolled cell cycle and apoptosis, which leads to the proliferation and migration of colorectal cancer cells. Moreover, whether CYP2S1 deficiency is also indirectly involved in inactivation of the p53 signaling pathway due to increased expression of Wnt16 remains to be explored. We also did not further verify whether CYP2S1 deficiency activates the Wnt signaling pathway to promote the occurrence and development of colorectal cancer.

Furthermore, other significantly upregulated genes also include MACC1, CYP24A1, CASP10, and TOMM34. The main cause of cancer death is cancer metastasis, Stein et al. reported that MACC1 is closely related to colorectal cancer metastasis and is a key gene that regulates cell proliferation, differentiation, and metastasis 37. MACC1 promotes the development of osteosarcoma by regulating the HGF/c-Met pathway and microtubule stability 38. MACC1 is abnormally increased in solid tumors such as colorectal cancer, pancreatic cancer and breast cancer 39-41. MACC1 promotes the development of colorectal cancer by activating the HGF/c-MET signaling pathway, which is consistent with the high expression of MACC1 in our sequencing results. It has been reported that mutant P53 is associated with hypomethylation of MACC1, leading to high expression of MACC1 and promoting the development of colorectal cancer 42. We can preliminarily assume that CYP2S1 knockout causes P53 mutation, MACC1 methylation decreases, and high MACC1 expression promotes the development of colorectal cancer. CYP24A1 has oncogenic potential in lung adenocarcinoma 43. CASP10 is involved in the diagnosis, prognosis, and progression of diverse cancer types 44. TOMM34 is involved in the growth of cancer cells, and may contribute to the development of novel anticancer drugs and/or diagnosis for CRC 45. However, further investigation into the deep mechanisms of CYP2S1 in tumors is needed. The upstream and downstream mechanisms of CYP2S1 in regulating metastasis and invasion need further investigation to inform clearer treatment strategies for CRC.

## Conclusion

In conclusion, the key point of this study is that knockout of CYP2S1 can promote the development of colorectal cancer. CYP2S1 knockout contributes to the entry of β-catenin into the nucleus of intestinal epithelial cells in *APC*^Min/+^ mice, and affects the Wnt signaling pathway, the p53 signaling pathway, and upregulates MACC1. It is a potential molecular target for the diagnosis and treatment of colorectal cancer.

## Supplementary Material

Supplementary figures and tables.

## Abbreviations

CRC: Colorectal cancer
CYP450: Cytochrome P450
CYP2S1: Cytochrome P450 Family 2 Subfamily S Member 1
*APC*: Adenomatosis polyposis coli
Min: Multiple intestinal neoplasia
MACC1: Metastasis associated in colon cancer 1
FAP: Familial adenomatous polyposis
MAPK: Mitogen-activated protein kinase
ERK: Extracellular regulated protein kinases
BRAF: B-Raf proto-oncogene, serine/threonine kinase
AHR: Aromatic hydrocarbon receptor
WT: Wild-type
H&E: Hematoxylin-eosin
IHC: Immunohistochemistry
PBS: Phosphate buffered saline
HRP: Horseradish peroxidase
IF: Immunofluorescence
DAB: Diaminobenzidine
WB: Western blot
SDS-PAGE: Sodium dodecyl sulfate‒polyacrylamide gel electrophoresis
TBST: Tris-buffered saline containing Tween-20
ECL: Enhanced chemiluminescence
PCR: Polymerase Chain Reaction
TCGA: The Cancer Genome Atlas
PECAM-1/CD31: Platelet endothelial cell adhesion molecule-1
EMT: Epithelial-to-mesenchymal transition
