# Supplementary material for: CYP2S1 Knockout Promotes Intestinal Tumor Growth in APCMin/+ Mice and Its Clinical Significance
Source: J Cancer. 2025 Jul 10;16(10):3128–40. doi: 10.7150/jca.111574 (PMC12305598; doi:10.7150/jca.111574)
Supplement: Supplementary file 1 — Supplementary figures and tables. [file jcav16p3128s1.pdf]

Supplementary Material

CYP2S1 Knockout Promates Intestinal Tumor Growth in APCMin/+ Mice and Reveals Its Clinical Significance

Yaqing Du[1#], Yunxia Kuang[1#], Xiuqiong Meng[1#], Bobing Zheng[1], Qinru Chen[1], Qian Yan[2], Jiangchao Li[1]*

#These authors contributed equally to this work.
1Laboratory of Oncology and Immunology, School of Basic Medical Sciences, Guangdong Pharmaceutical University, Guangzhou, 510006, China.
2Guangdong Institute of Gastroenterology, Guangdong Provincial Key Laboratory of Colorectal and Pelvic Floor Diseases, The Sixth Affiliated Hospital, Sun Yat-sen University, Room 703, Guangzhou, 510006, China.

***Corresponding author**
Dr. Jiangchao Li
Laboratory of Oncology and Immunology, School of Basic Medical Sciences, Guangdong Pharmaceutical University, Guangzhou, 510006, China.
Address: No. 280 Waihuan Rd. E, Higher Education Mega Center, Guangzhou 510006, China.
Office Phone: 86-20-39352126; E-mail: lijiangchao@gdpu.edu.cn

Supplement Table 1 The primer sequences of qPCR

Gene Name	Forward Primer Sequence (5'-3')	Gene Name	Reverse Primer Sequence (5'-3')
H-GAPDH-F	GGACCTGACCTGCCGTCTAG	H-GAPDH-R	GTAGCCCAGGATGCCCTTGA
H-CYP2S1-F	GATGCTGGAAGGGACTTTTG	H-CYP2S1-R	GATCAGCTCCTCGCCTTCT
H-β-actin F	CTGGAACGGTGAAGGTGACA	H-β-actin R	AAGGGACTTCCTGTAACAACGCA

The primer table for genotype identification of mice.

Gene Name	Sequence(5'-3')	PRIMER TYPE
M-CYP2S1-F1	TCTCTAAATAAGAGGGTAGTGGGC	Mutant Forward
M-CYP2S1-F2	AACTTGCTGATGACGGTCACATA	Wild type Forward
M-CYP2S1-R	GGTGCTAACTGGGAATGTTACCC	Common type
M-*APC*-F1	TTCTGAGAAAGACAGAATTA	Mutant Forward
M-*APC*-F2	GCCATCCCTTCACGTTAG	Wild type Forward
M-*APC*-R	TTCCACTTTGGCTAAGGC	Common type

H=Human M=mouse.

Supplement Table 2 The siRNA sequences

Name	Forward Primer Sequence (5'-3')
si-NC	UUCUCCGAACGUGUCACGUTTACGUGACACGUUCGGAGAATT
si-CYP2S1-1	GCUGAUGACAGUCAUUUAUTTAUAAAUGACUGUCAUCAGCTT
si-CYP2S1-2	CAGCUGAGGAAGUUUACCATTUGGUAAACUUCCUCAGCUGTT

Supplement Table 3 The list of antibodies

Name	Company	Cat number
β-catenin	BD	610153
CYP2S1	HUABIO	ER63173
Bax	UpingBio	YP-Ab-00317
Bcl-2	UpingBio	YP-Ab-00322
Caspase3	UpingBio	YP-Ab-00345
Cleaved caspase3	UpingBio	YP-Ab-00003
E-cadherin	CST	3195S
N-cadherin	CST	13116S
Vimentin	BOSTER	BM0135
GAPDH	CST	5174S
CTNNB1	BOSTER	BM1766
HRP-Anti-rabbit IgG	ZSGB-BIO	ZB-2301
HRP-Anti-mouse IgG	ZSGB-BIO	ZB-2305
CD31	Abcam	ab28364
Ki67	Abcam	ab16667
Goat anti-Mouse-488	Invitrogen	A-11001

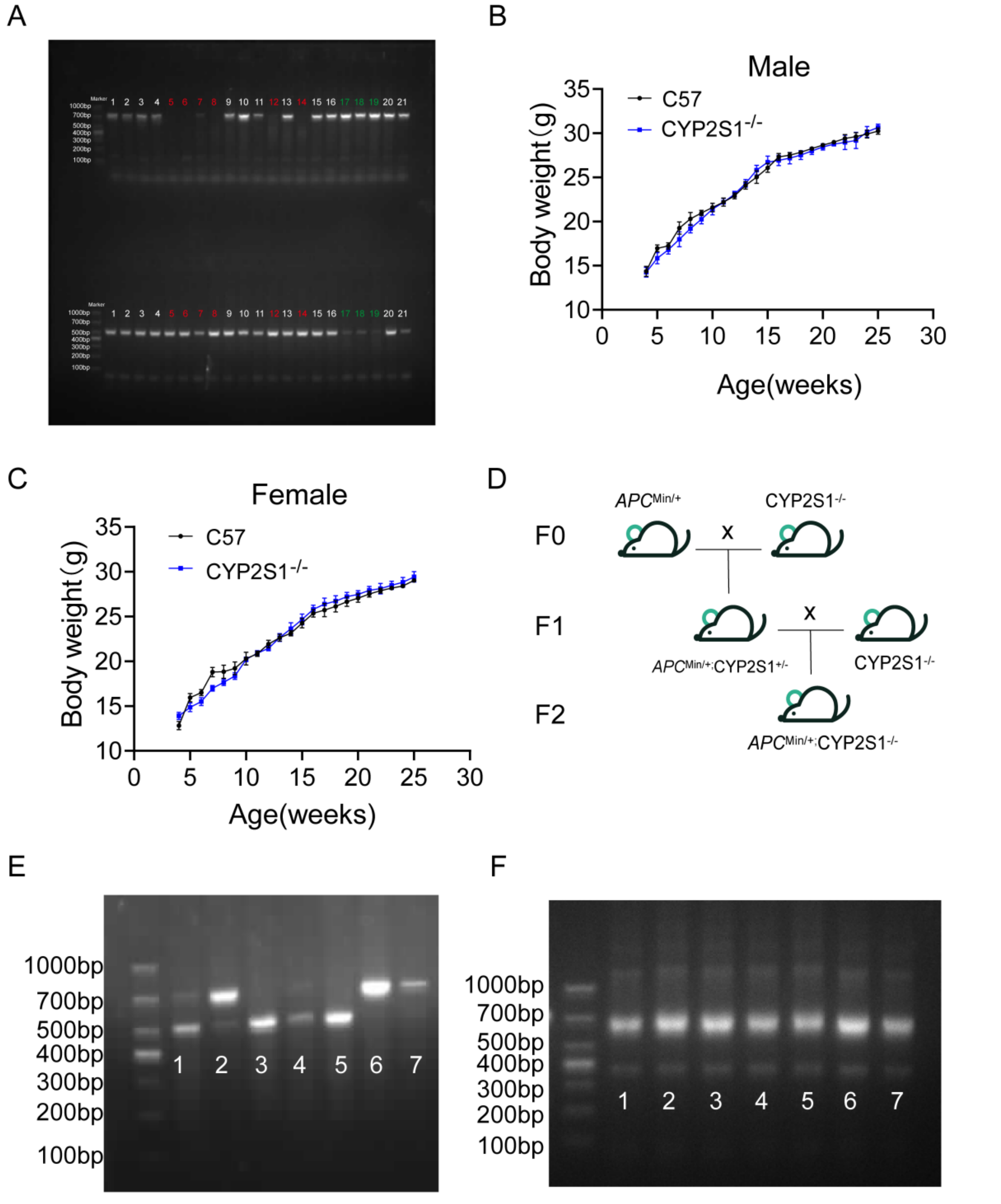

Figure S1 Generation and Genotyping of *APC*$^{Min/+}$;CYP2S1$^{-/-}$ mice

(A) The results of CYP2S1 genotype identification in mice (n = 21).The CYP2S1 knockout mouse PCR production size was 514 bp (#5-8, #12 and 14), and the wild-type mouse PCR production is at 717 bp (#17-19). CYP2S1$^{+/-}$ with both 717 bp and 514 bp(#1-4, #9-11, #13, #15-16 and #20-21). (B and C) Body weight changes in male and female CYP2S1 knockout mice from 4 to 25 weeks of age. (D) Generation of *APC*$^{Min/+}$;CYP2S1$^{-/-}$ mice. (E) Genotyping of CYP2S1$^{-/-}$ in *APC*$^{Min/+}$; CYP2S1$^{-/-}$ mice (n = 7).CYP2S1 knockout mouse PCR production size was 514 bp.(F) Genotyping of *APC*$^{Min/+}$ genotype in *APC*$^{Min/+}$; CYP2S1$^{-/-}$ mice (n = 7).*APC*$^{Min/+}$ knockout mouse PCR production size was 340bp and 600bp.

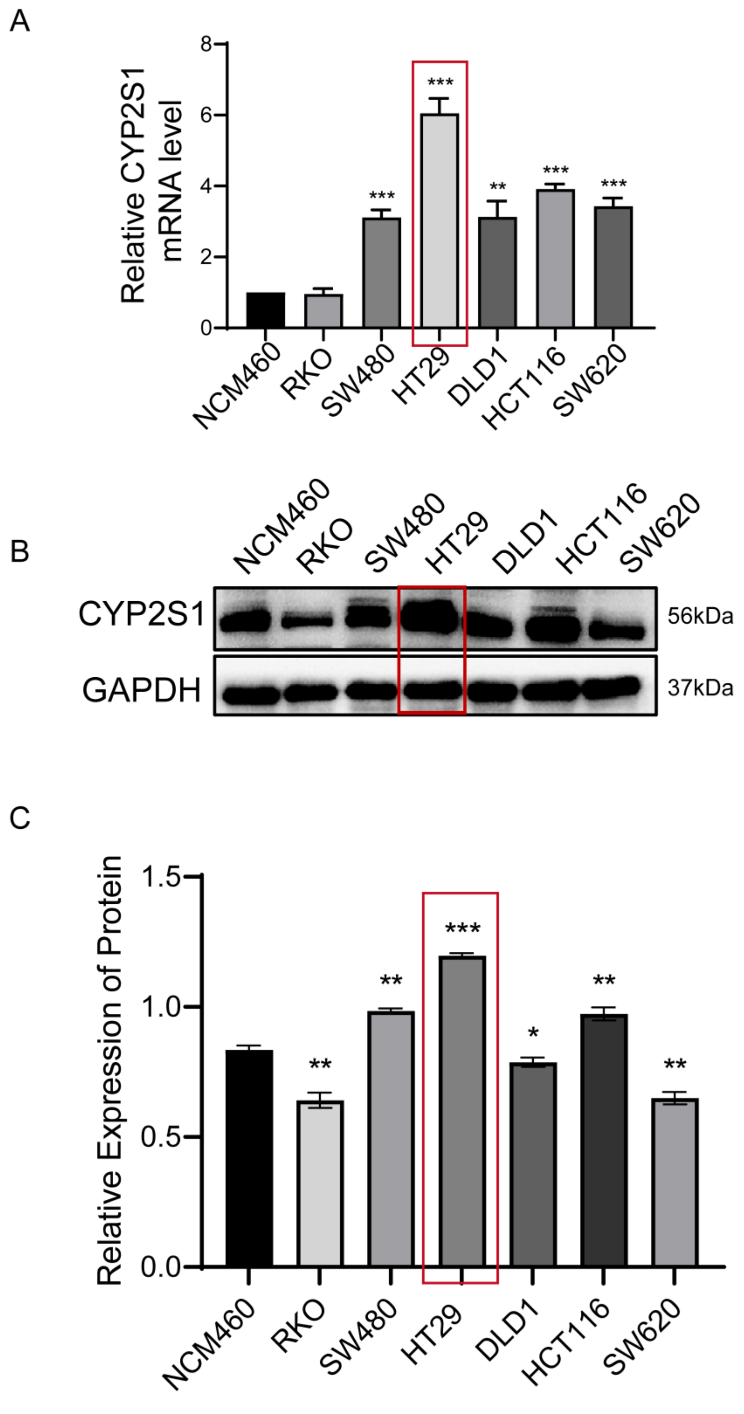

Figure S2 Expression of CYP2S1 mRNA and protein in various colorectal cancer cell lines
(A) Relative gene expression levels in colorectal cancer cell lines compared to NCM460, normalized to β-actin (***P* < 0.01 ; ****P* < 0.001). (B) CYP2S1 expression in CRC cell lines assessed by Western blot, normalized to GAPDH. (C) Protein expression levels were significantly different based on quantitative analysis (**P* < 0.05; ***P* < 0.01; ****P* < 0.001).

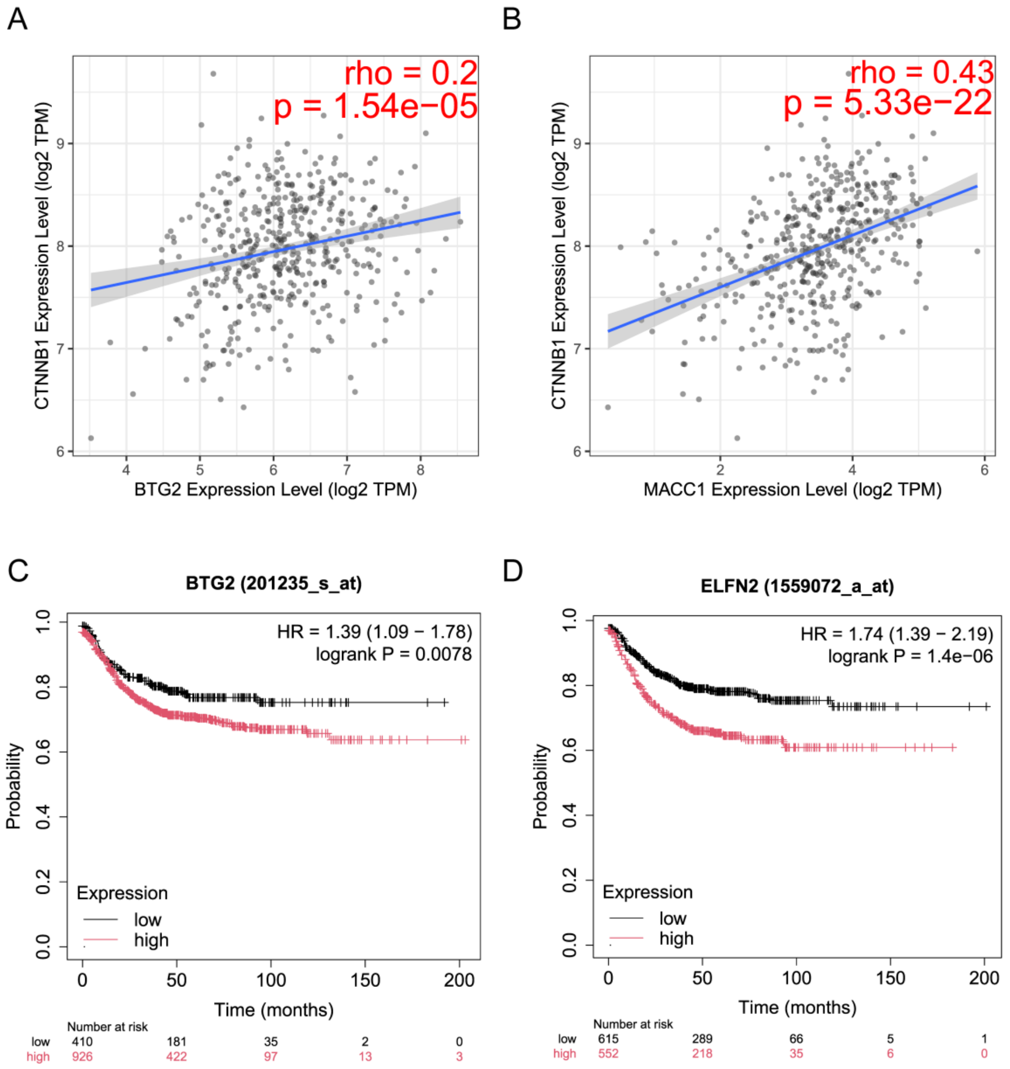

Figure S3 Correlation analysis of BTG2 and MACC1 with CTNNB1,and Kaplan-Meier survival curve analysis for BTG2 and ELFN2

(A and B) CTNNB1 (coding beta-catenin) was positively correlated with BTG2 (rho = 0.2,*P* = 1.54e-05) and MACC1(rho = 0.43,*P* = 5.33e-22) in the TCGA-CRC database. (C and D) Kaplan-Meier survival analysis showed that high expression levels of BTG2 and ELFN2 were significantly associated with poor prognosis in colorectal cancer patients.

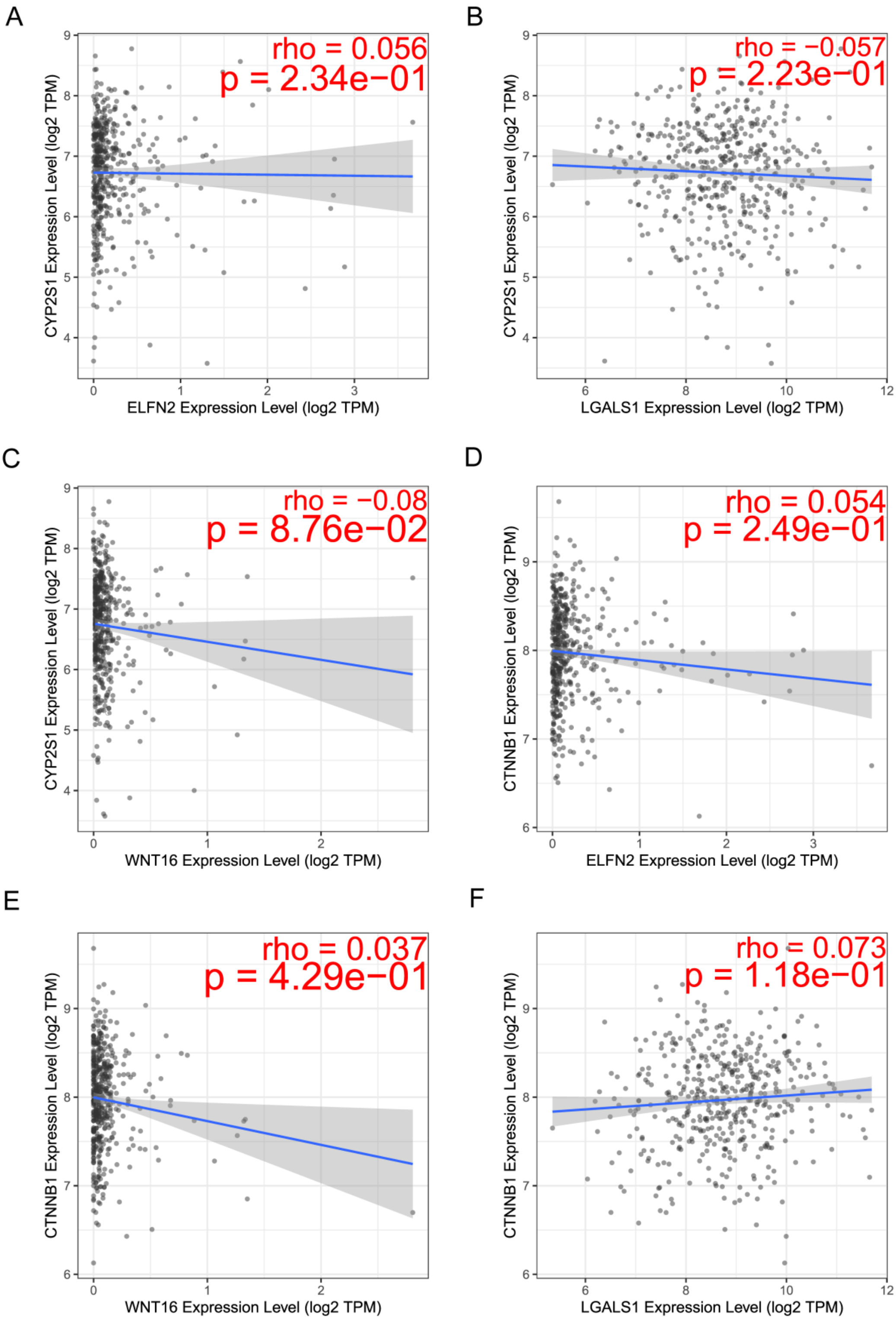

Figure S4 Some **s**ignificantly changed genes showed no statistically significant correlation with either CYP2S1 or CTNNB1

(A-C) In the sequencing data of colorectal cancer patients, ELFN2 (rho = 0.056,*P* = 2.34e-01) was positively correlated with CYP2S1, while, LGALS1 (rho = -0.057,*P* = 2.23e-01)and WNT16 (rho = -

0.08,$P = 8.76\text{e-}02$) were negatively correlated with CYP2S1. (D-F) The expression levels of ELFN2 (rho = 0.054,$P = 2.49\text{e-}01$), WNT16 (rho = 0.037,$P = 4.29\text{e-}01$) and LGALS1 (rho = 0.073,$P = 1.18\text{e-}01$) showed positively correlated with CTNNB1 in colorectal cancer patients.

A

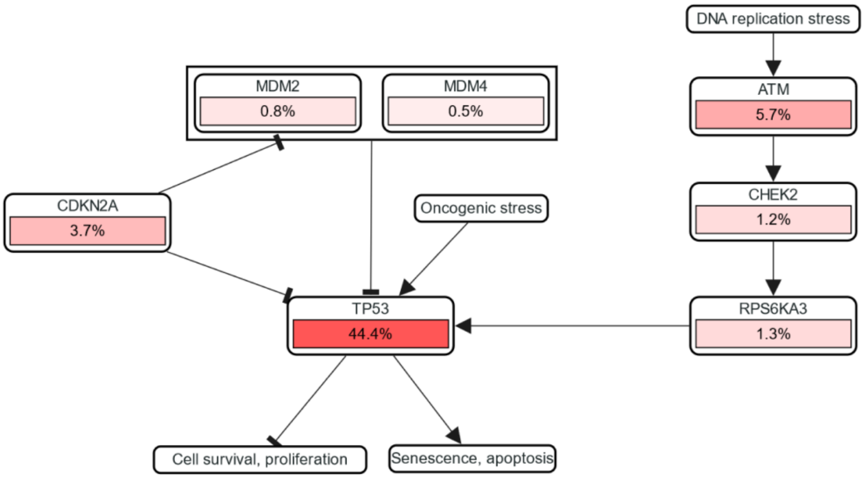

B

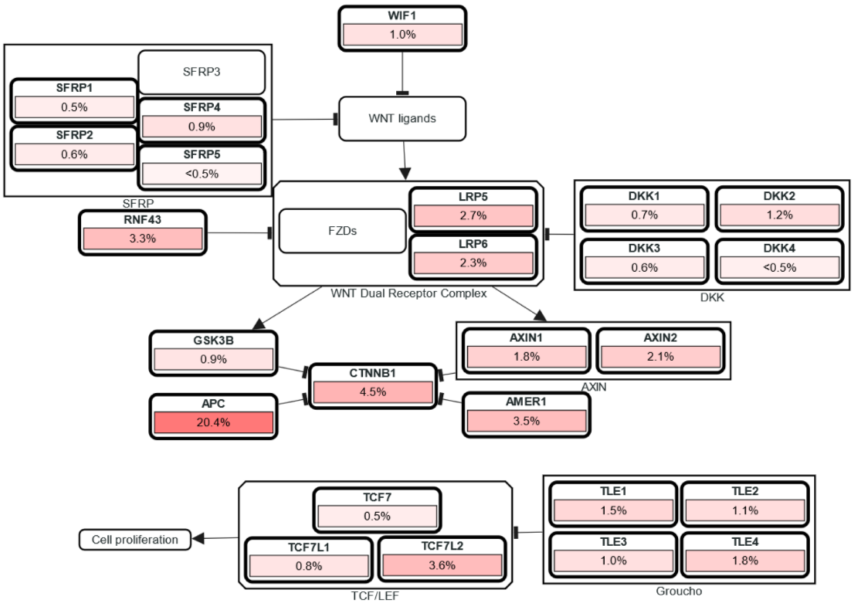

Figure S5 The frequency of alterations in the p53 and *APC*-β-catenin signaling pathways is high in colorectal cancer were high

(A) TP53 mutations were present in 44.4%. (B)*APC* mutations in 20.4%, and CTNNB1 mutations in 4.5%.

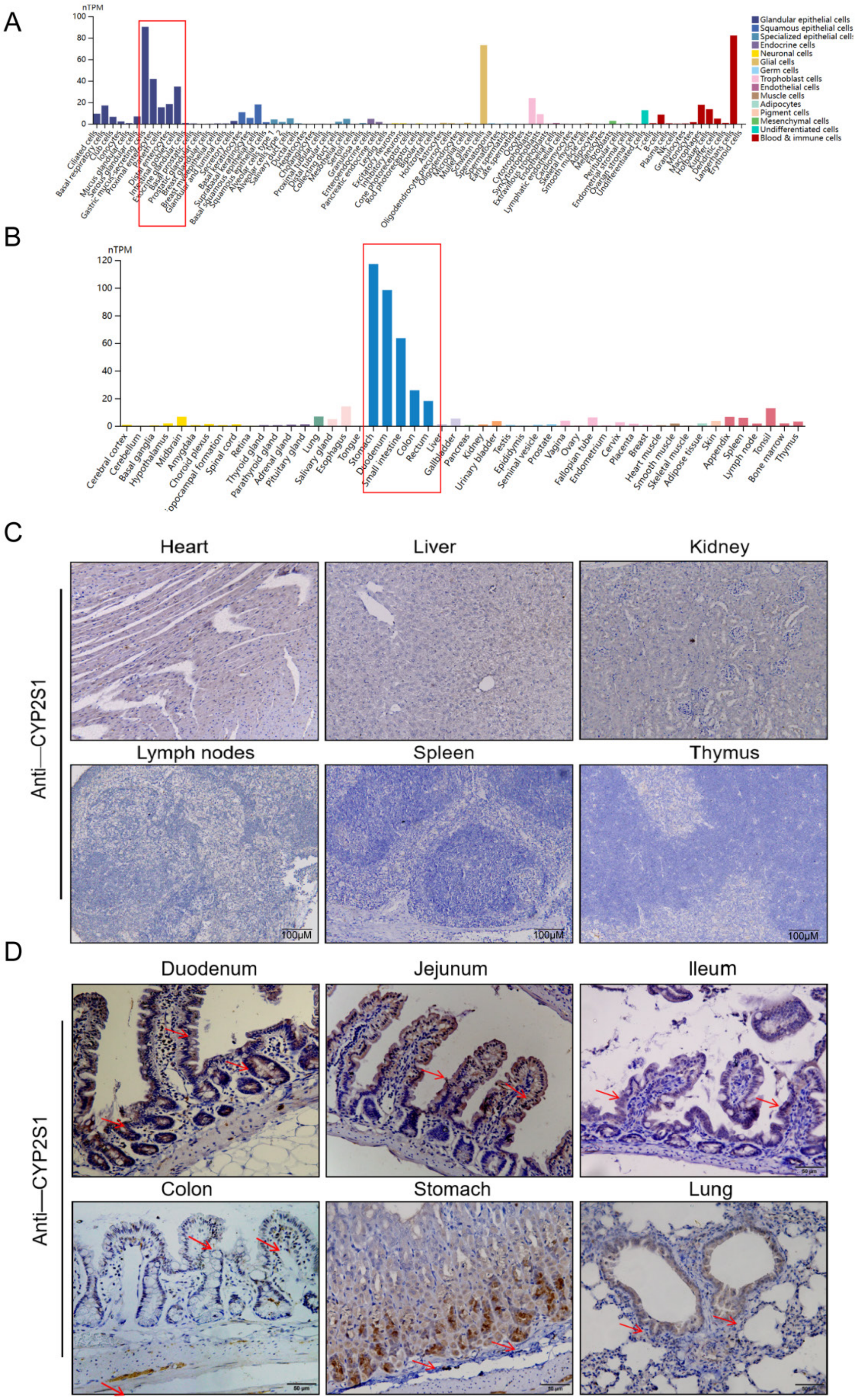

Figure S6 High expression of CYP2S1 in the digestive tract tissues of both humans and mice

(A and B) Expression of CYP2S1 in human cells and organs tissues data derived from the Human Protein Atlas. (C and D) Immunohistochemical staining of the expression and distribution of CYP2S1 protein in various mouse tissues. CYP2S1 showed relatively higher expression levels in digestive tract tissues

concluding duodenum,jejunum,ileun,colon and stomach（scale bar = 50 μM).
